# Colony-Level Differences in the Scaling Rules Governing Wood Ant Compound Eye Structure

**DOI:** 10.1038/srep24204

**Published:** 2016-04-12

**Authors:** Craig D. Perl, Jeremy E. Niven

**Affiliations:** 1 School of Life Sciences and Centre for Computational Neuroscience and Robotics, University of Sussex, Falmer, Brighton, BN1 9QG, UK

## Abstract

Differential organ growth during development is essential for adults to maintain the correct proportions and achieve their characteristic shape. Organs scale with body size, a process known as allometry that has been studied extensively in a range of organisms. Such scaling rules, typically studied from a limited sample, are assumed to apply to all members of a population and/or species. Here we study scaling in the compound eyes of workers of the wood ant, *Formica rufa*, from different colonies within a single population. Workers’ eye area increased with body size in all the colonies showing a negative allometry. However, both the slope and intercept of some allometric scaling relationships differed significantly among colonies. Moreover, though mean facet diameter and facet number increased with body size, some colonies primarily increased facet number whereas others increased facet diameter, showing that the cellular level processes underlying organ scaling differed among colonies. Thus, the rules that govern scaling at the organ and cellular levels can differ even within a single population.

Understanding how organ size and shape is controlled during development is a major challenge in biology. The control of organ morphology is particularly problematic for organisms that need to develop organs to meet specific requirements under fluctuating conditions and resources. In natural environments, adults from a single species can vary enormously in body size due to a combination of genetic and environmental factors. The changes in organ size that accompany changes in body size can be characterised by allometric scaling relationships. The scaling of any feature with size can be described by:


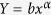


where *x* is a measure of body size, *Y* is the size of the organ in question, 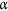 is the scaling exponent and *b* is the initial growth index^1^.

When no change occurs in the relative size of an organ with body size (*α* = 1) the relationship is described as isometric^2^. More typically, however, organs show negative allometries (*α* < 1) becoming relatively smaller as body size increases^3^,^4^. Even with negative allometries organs can be absolutely larger in animals with a greater body size, but relatively smaller when compared to smaller conspecifics^1^,^5^,^6^. In rare cases, organ size may show positive allometry increasing relatively faster than body size (*α* > 1)^7^,^8^. Such positive allometry is often associated with organs under sexual selection^7^,^8^.

The scaling of different organs within a body is the product of differential growth; as an organism grows larger, certain organs grow at a faster rate than others^3^,^9^. This is thought to occur through differential resource allocation^9^,^10^,^11^, whereby resources are distributed to different organs at different rates. Scaling has been studied in many taxa including mammals and birds^12^,^13^,^14^,^15^ and especially insects^15^,^16^,^17^,^18^,^19^. In part, this is due to the power of genetic tools available in the fruit fly *Drosophila melanogaster*^20^ but also because of the mode of development of holometabolous insects: the organs of these adult insects develop at the end of a period of larval growth from ectodermally-derived cellular monolayers called imaginal discs^21^,^22^.

Differential resource allocation to imaginal discs during pupation is mediated through insulin-like peptides (ILPs) and their receptors^23^. During the larval (or feeding) stage ILPs are produced in response to changes in nutrition^24^,^25^ and, along with ecdysone, are responsible for inducing somatic growth^26^. During the pupal (or non-feeding) stage imaginal disc cell growth is also mediated by ILPs, but ILP release is controlled via ecdysone levels instead of responding to nutrition^26^. Insulin receptors are expressed by imaginal disc cells^27^; the greater the number of receptors, the more sensitive the disc is to increases in ILPs^9^. Hence, greater nutrition leads to increases in organ size, but the scaling of different organs varies depending on the relative sensitivities to ILPs^28^,^29^,^30^. Additional factors, including genetics^20^,^31^,^32^ and temperature^20^,^25^, will also have an impact upon scaling and size changes in response to feeding.

Organs such as the compound eyes and wings of insects provide an opportunity to explore scaling at the cellular level because external structures visible in adult organs provide a read-out at a cellular-level resolution^9^,^10^. In compound eyes this means that the size of the facets are representative of the level of cellular growth and division that occurs during development^33^. The scaling of compound eyes with body size has been investigated in numerous insect species^5^,^34^,^35^,^36^,^37^,^38^,^39^,^40^,^41^. In all these investigations insect compound eyes increase in size (measured as either eye length or area) with increasing body size but show negative allometry. Some species, such as *Cataglyphis albicans*^37^*, C. bicolor*^37^*, C. fortis*^37^, *Camponotus pennsylvanicus*^5^ and *Melophorus bagoti*^38^, primarily increase facet number as they get larger whereas others, such as *Bombus terrestris*^39^,^40^ and *Solenopsis* sp.^41^, and *Formica integroides*^34^ increase facet diameter and facet number.

Wood ants, *Formica rufa* (L.), form nest mounds (Fig. 1a) containing up to 100 queens and 100,000–400,000 workers without distinct castes^42^. Workers form large trails within woodlands (Fig. 1b) and use visually-guided navigation whilst foraging^43^, ensuring that resource allocation to the compound eye is important for their ecology, and suggesting that scaling relationships within the visual system have functional consequences. We studied the scaling of wood ant compound eyes, exploring organ-level morphological changes in eye area, facet number and size and how this differed between nests. We found substantial heterogeneity in eye scaling between different nests within the same population. This heterogeneity calls into question many inherent assumptions made by studies examining differential organ scaling.

## Results

### Size variation in wood ants

**Figure 1:**
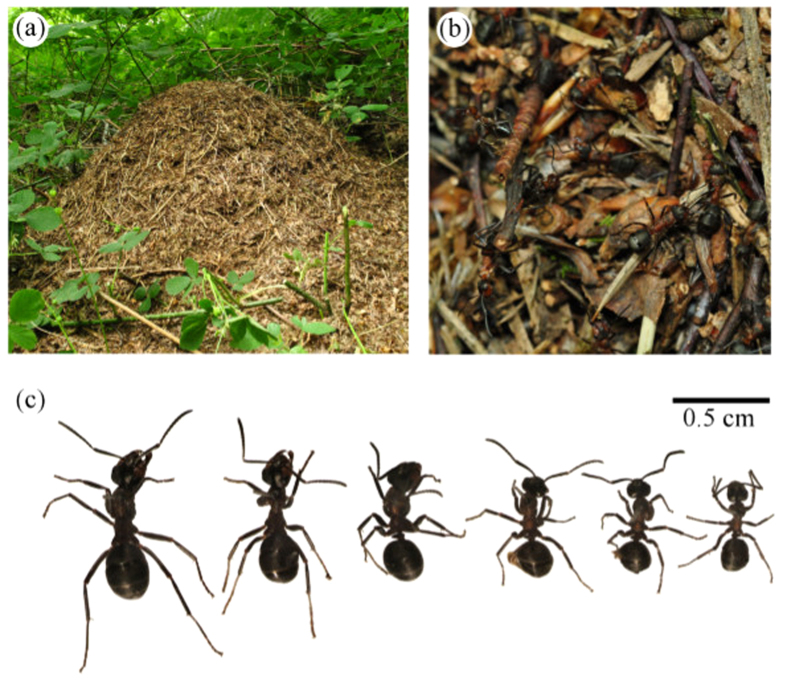
Size variation in wood ant (*Formica rufa*) workers. (**a**) A wood ant nest, and (**b**) workers on a foraging trail. (**c**) Workers from a single nest are morphologically undifferentiated but span a wide range of body sizes.

**Figure 2:**
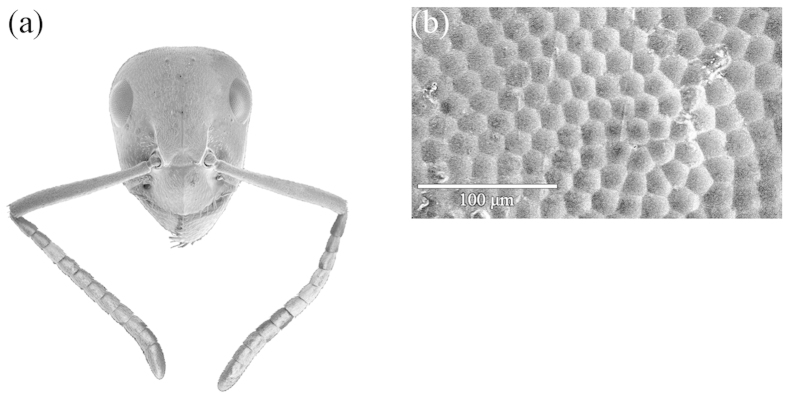
Facets in the compound eye of a red wood ant. (**a**) A frontal view of the head of a large worker viewed under a scanning electron microscope. (**b**) Close-up of surface of worker eye showing details of the facet array.

Wood ant workers lack distinct morphological castes but they span a wide range of body sizes (Fig. 1c). Even within a single colony, the smallest workers can be less than half the size of the largest (Figs 1c and S1). Irrespective of size, workers possess compound eyes located dorso-laterally on their head (Fig. 2). These eyes are smaller and flatter than of the majority of the compound eyes of other insects whose visual ecology is studied *e.g*.^35^. Larger workers possess larger eyes than their smaller counterparts (Fig. S2).

### Eye morphology

**Figure 3:**
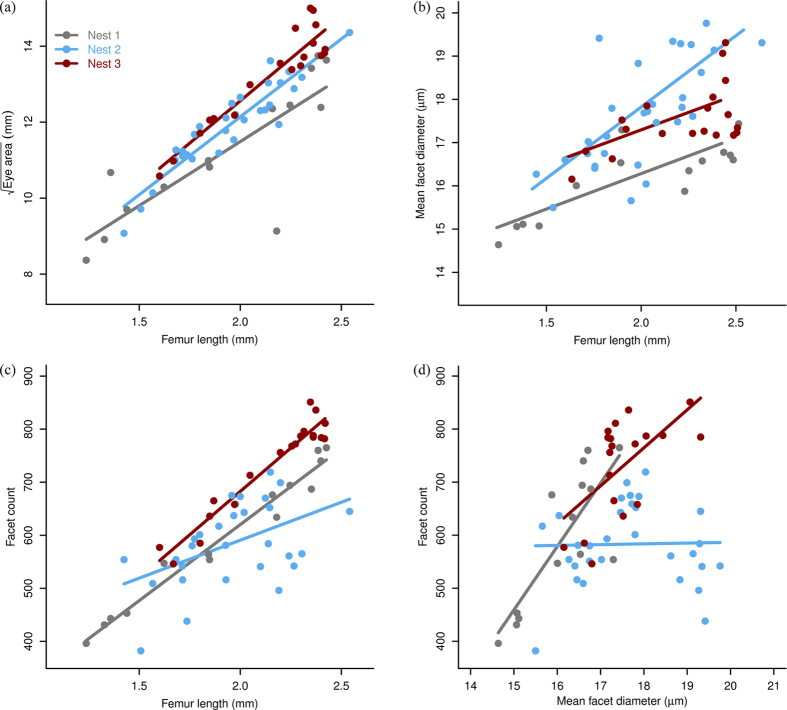
Differential scaling of compound eyes and facets among colonies. Allometric scaling of (**a**) eye √area (mm) (r^2^ values: Nest #1: 0.66; Nest #2: 0.86; Nest #3: 0.87), (**b**) mean facet diameter (μm) (r^2^ values: Nest #1: 0.66; Nest #2: 0.46; Nest #3: 0.28) and (**c**) facet number with a proxy of body size (femur length) (r^2^ values: Nest #1: 0.97; Nest #2: 0.21; Nest #3: 0.93). (**d**) Scaling of facet number with mean facet diameter (r^2^ values: Nest #1: 0.63; Nest #2: −0.04; Nest #3: 0.32). Sample sizes: Nest #1: 17; Nest #2: 29; Nest #3: 19.

We quantified the differences in the area of compound eyes of small and large workers from three separate nests, using the square-root of eye area to preserve dimensionality (Fig. 3). As expected, in all three nests the compound eye area increased with increasing ant size (F_63,59_ = 297.16, p < 0.001; subscript denotes sample size, degrees of freedom). The compound eyes of smaller workers were absolutely smaller but relatively larger than those of their larger counterparts (Fig. 3a). Consequently, for each of the three nests eye area had a negative allometric scaling relationship with hind femur length^44^, which we used as a proxy for body size (Table S1). Comparisons among the three nests showed significant differences in mean eye area (F_63,59_ = 12.25, p < 0.001) but failed to reveal a significant interaction between eye area and body size (F_63,57_ = 1.54, p = 0.22), indicating that the rate of increase in eye area with body size is similar among nests. Pairwise comparisons between nests revealed that the mean eye area of ants from nest #1 differed from that of nest #2 (t_63,59_ = 2.95, p < 0.01) and #3 (t_63,59_ = 4.91, p < 0.001). The mean eye area of ants from nest #2 also differed from that of nest #3 (t_63,59_ = 2.74, p < 0.01).

We assessed the differences in eye area scaling among nests using principle component analysis (PCA) followed by cluster analysis. PCA was used to reduce the three variables of interest (femur length, eye area and nest) to two principle components. The first two principle components explained 97% of the variation in the data. Principle component 1 (PC1) was negatively correlated with all three variables, though primarily femur length and eye area, whereas PC2 was strongly positively correlated with nest affiliation (Table S2 and Fig. S3). Subsequent cluster analysis revealed that there were four clusters; one corresponding to nest #1, another for nest #2, and a further two clusters for points belonging to nest #3 (Table S2 and Fig. S3).

Changes in both the diameter and number of facets could account for the scaling of eye area with body size, and for the differential scaling of eye area among nests. We measured the diameter of facets from the compound eyes of small and large workers from all three nests. The diameter of every facet from a representative and small and large ant were measured, yielding facet ranges of 12.45–21.51 μm and 15.33–23.22 μm, respectively. Our measurements showed that mean facet diameters are larger in larger ants (F_63,59_ = 50.91, p < 0.001) (Fig. 3b). However, mean facet diameter was relatively larger in small ants compared with their larger counterparts. Consequently, for each of the three nests facet diameter had a negative allometric scaling relationship (Table S1). Comparison among the three nests showed significant differences in their mean facet diameter (F_63,59_ = 15.89, p < 0.001). Ants from nest #1 had significantly smaller mean facet diameters than those from nest #2 (t_63,59_ = 5.57, p < 0.001) or nest #3 (t_63,59_ = 3.40, p < 0.01). Ants from nest #3 had significantly larger facet diameters than those from nest #2 (t_63,59_ = 2.31, p = 0.02). Yet despite the differences in mean facet diameter, the rate of increase in facet diameter with body size did not differ between nests (F_63,57_ = 3.13, p = 0.05).

We also counted all the facets from the compound eyes of each worker from which we had previously measured the area and facet diameters. As expected, the number of facets per eye increased with body size (F_63,57_ = 206.27, p < 0.001) (Fig. 3c). Our counts revealed that smaller ants had relatively more facets than their larger counterparts and, akin to area and facet diameter, facet number had a negative allometric scaling relationship for each of the three nests (Table S1). Comparisons among nests revealed differences in the mean number of facets (F_63,57_ = 20.58, p < 0.001). Ants from nest #2 had fewer facets per eye than those from nest #1 (t_63,57_ = 2.73, p < 0.01) or nest #3 (t_63,57_ = 2.40, p = 0.02). Ants from nest #1 and nest #3 did not differ in their mean number of facets per eye (t_63_,_57_ = 0.16, p = 0.87). Comparisons among all three nests also revealed a significant interaction between the rate of increase in facet number and body size (F_63,57_ = 6.56, p < 0.01), indicating that it differs for ants from different nests. Pairwise comparisons revealed that the rate of increase in facet number did not differ in ants from nests #1 and #3 (t_63,57_ = 0.73, p = 0.46) but that ants from nest #2 showed a slower rate of increase in facet number than either nest #1 (t_63_,_57_ = 2.99, p < 0.01) or #3 (t_63,57_ = 3.23, p < 0.01).

To establish how facet diameter and facet number contributed to eye area for each nest, we examined how facet number increased as a function of mean facet diameter (Fig. 3d). Across all three nests combined, there was a significant increase in facet number with larger mean facet diameters (F_63,57,_ = 12.19, p < 0.001). A significant interaction term between mean facet diameter and nest (F_63,57_ = 22.25, p < 0.001) indicated that the rate of facet number increase with increasing facet diameter is different for ants from different nests. Pairwise comparisons revealed that the rate at which facet number increased differed between nests #1 and #2 (t_63,57_ = 4.35, p < 0.001) and nests #2 and #3 (t_63,57_ = 2.67, p < 0.01), though not between nests #1 and #3 (t_63,57_ = 1.40, p > 0.1).

Visual examination of the data indicated a subset of putative outliers (Fig. 3d). We used PCA combined with cluster analysis to investigate whether these ants formed a distinct group of individuals following different scaling rules from the remainder of nest #2. Again, PCA was used to reduce the three variables of interest (facet number, mean facet diameter and nest affiliation) to two principle components. The first two principle components explained 82.7% of the variance in the data. Principle component 1 (PC1) was negatively correlated with all three variables to largely equal extents, whereas PC2 was strongly negatively correlated with mean facet diameter (Table S2). The cluster analysis revealed three clusters (Fig. S4). One cluster was formed from representatives of all three nests and another from ants exclusively from nest #2. There was also a third cluster composed of ants exclusively from nest #3, though there were no obvious outliers (Fig. S4). This independent nest #3 cluster is formed from ants that have a facet count higher than predicted from the regression line. The independent nest #2 cluster is formed from the individuals that we identified as putative outliers from nest #2.

## Discussion

By making use of the unique structure of the insect compound eye, we were able to analyse scaling rules that govern organ size. These rules differ among nests from the same population, as well as differing between ants from the same nest. Below we discuss the causes and consequences of these differences in scaling, and the implications for scaling studies more generally.

As wood ant workers’ body size increases, so too does the area of their compound eyes, as well as the numbers of facets and their diameters, though they do so with negative allometry. Consequently, smaller ants have compound eyes with relatively larger areas and facet diameters, and relatively more facets than their larger counterparts. These scaling relationships occur in all the nests we studied and, in this respect, they resemble relationships observed in other insect species such as *Formica integroides*^34^, *Cataglyphis* sp.^37^, *Melophorus bagoti*^38^, *Bombus terrestris*^39^ and *Solenopsis* sp.^41^. However, comparison among nests reveals significant differences in their scaling relationships, more typical of those reported among species. Both grade shifts and slope shifts occur depending upon the specific parameter measured. The scaling of eye area primarily differs in intercept among nests, characteristic of grade shifts. Indeed, ants from all three nests differed from each other in terms of eye area. Differences also occurred at the cellular level: mean facet diameters show grade shifts among all three nests; both slope shifts and grade shifts occur in facet number among nests; and both grade shifts and slope shifts occur when facet number scales against mean facet diameter.

Consequently, formulating definitive rules about the allometric scaling of wood ant compound eyes is difficult because no two nests followed similar patterns. Rather than increases in eye area being mediated through either increased facet numbers or diameters, both contribute in a nest-dependent manner. Patterns of eye growth are further complicated by subsets of ants from a given nest using different scaling rules to govern the development of their eyes; ants from nest #2 and #3 contained individuals with a different relationship between facet diameter and number compared with the majority of the sampled population. Thus, there is considerable plasticity in scaling rules across wood ant populations to which both genetic and environmental factors may contribute.

The nests we compared were all from the same polygynous population, and are likely to have been closely related because polygynous *Formica* sp. alates do not disperse far^45^. Nevertheless, there may be substantial genetic variability within the nests because workers may be the progeny of up to 100 queens, and may not be true sisters at all due to polyandry^44^. Thus, genetic factors, which are known to affect scaling relationships^20^,^31^,^45^,^46^, may contribute to scaling differences. Despite being derived from the same locale, the nests may have been subject to different environmental conditions, including nutrition and temperature, which could contribute to differences in scaling relationships. Larval nutrition influences adult body size in insects, with greater access to nutrition giving rise to larger adults^47^,^48^. Temperature likewise affects the growth of insect larvae because they are ectothermic, faster growth in warmer conditions typically resulting in relatively smaller adults^49^,^50^. Both temperature and nutrition influence organ scaling in fruit flies^20^, which like ants are holometabolous, suggesting that these factors may affect scaling.

Nutritional differences among wood ant nests may arise because, following territorial skirmishes at the beginning of the season^51^,^52^, the trees that they have access to vary in the numbers of aphids from which honeydew can be obtained and other invertebrates (for protein) that they host. This will produce differences in larval nutrition, influencing their growth and, consequently, resource allocation to developing organs^8^. Differences in nutrition could, therefore, partially explain differences in scaling relationships among wood ant nests.

Temperature differences and fluctuations are also common in natural environments, though *F. rufa* group ants attempt to maintain constant nest temperatures through various mechanisms including site selection to ensure direct access to solar radiation, metabolic heat generation by workers, and from decomposing plant material in larger nests^53^,^54^,^55^.Wood ant workers also move larvae within the nest, placing them in different thermal environments during development^53^. This suggests that, to some extent, wood ants can compensate for temperature differences and fluctuations within the local environment, though the effectiveness of this buffering is unknown.

Wood ant nests differ not only in the scaling of relative organ size but also in the cellular-level rules from which the organs are constructed, so that in some nests larger eyes are primarily composed of more facets whereas in others they are primarily composed of larger facets. Nests #1 and #3 show increases in both facet number and diameter, implying organ scaling through increases in cell size and number, a phenomenon also described in *Drosophila melanogaster*^32^. Current models of organ growth offer a proximate explanation for such differences. During the non-feeding stage of holometabolous larvae, levels of insulin-like peptides (ILPs) and ecdysone control cell proliferation and growth, respectively, in imaginal discs^24^,^25^,^26^. The release of these hormones is linked with nutrition during the larval feeding stage^25^,^26^. Thus, increases in facet number may be due to relatively greater levels of ecdysone and increases in facet diameter due to relatively greater levels of ILPs. The genetic background may interact with environmental factors^45^, which can themselves interact, to influence the extent of cellular proliferation or growth resulting in more or larger facets.

Both the number of facets within the compound eye and their diameters affect vision. Increases in facet number provide greater spatial resolution by increasing sampling of the visual field whilst increases in facet diameter improve sensitivity through increased photon capture^56^. The putative trade-off between increasing facet number and increasing facet diameter implies that nests are engaging in different developmental processes, investing in different aspects of vision.

The rules that govern the scaling of organs are often assumed to be a fundamental characteristic of a particular class of organism (*e.g.* species, sex). Typically, small numbers of organisms from single populations are used to determine the scaling of a particular trait with the assumption that the entire class conforms to the same relationship^57^,^58^,^59^. Our study suggests that this assumption does not always hold true. For *F. rufa*, there was considerable variation in allometric scaling relationships even among nests within the same population. Furthermore, allometric scaling studies often focus on the organ level^3^,^7^,^8^, ignoring the cellular level. Our study shows that the structure of organs may vary considerably at the cellular level, changes in organ size being produced by a combination of cell size and number. Our results provide a strong impetus for further investigations examining the interplay of cellular division and growth on the allometry of whole organs, and how these are affected by changes in nutrition and other environmental conditions. Together, our findings emphasise that allometric scaling relationships are highly malleable, at the organ and cellular levels, such malleability presumably allowing organisms to adapt their form to prevailing environmental conditions.

## Methods

### Animals

Whole colonies of *Formica rufa* (L.) (Hymenoptera: Formicidae) were collected from Ashdown Forest, Sussex (N 51 4.680, E 0 1.800) between June 2013 and August 2014, and maintained under a 12:12 hour light:dark cycle indoors at 21 °C. Foraging workers from nest #1 and #2 were sampled simultaneously during the end of 2013, ants from nest #3 were sampled from August 2014.

### Specimen preparation

Individual ants were selected from a colony at random and restrained with Plasticine (Early Learning Centre, UK). Transparent nail varnish (Rimmel London, UK) was applied to both compound eyes using a cocktail stick to create a cast. Ants were then stored at 4 °C for a minimum of 48 hours to ensure the casts completely dried. The nail varnish casts were removed, flattened with incisions and mounted on to 12.5 mm specimen stubs (Agar Scientific, UK). Casts were made as in Ribi *et al.*^60^. The rear left femur of each ant was mounted along with the eye cast as a proxy for the size of the ant. Nail-varnish eye casts and femurs were gold-coated and imaged using a scanning electron microscope (S420 Stereoscan, LEO Electron Microscopy Ltd., Germany).

### Measurements

Sixty six ants from three separate colonies were measured; 17 from nest #1, 30 from nest #2 and 19 from nest #3. Femur length, facet diameters, facet counts, eye areas and eye dimensions were measured from scanning electron micrographs using ImageJ v.1.48^61^. To account for variation in facet diameters across the compound eye, we split the eye into four regions (anterior, posterior, dorsal and ventral). Facet diameters were measured from three sets of three facets from these eye regions (*i.e.* 3 measurements from each region, 12 measurements from each cast). In two ants, the diameters of all the facets were measured. Eye area was calculated by approximating the shape of the eye as an oval. To validate this approximation we measured the real eye area from 15 ants (five ants per nest) and compared the real measurements with the approximations using a linear regression (Fig. S5). The ovals provided an accurate measure of eye area (intercept = 4.39 ± 5.62, p = 0.448; slope = 0.96 ± 0.03, p < 0.0001; r^2^ = 0.98). Facets were counted by hand from one of the eyes of each ant from scanning electron micrographs. Femur length was selected as a proxy for body size (though it scales positively with body size in Formicines^44^).

### Statistics

Eye area, mean facet diameter and facet number were analysed using analysis of covariance (ANCOVA) constructed with R base-package^62^. Non-significant ANCOVA terms were eliminated step-wise until only significant terms remained in the model. For cases in which data violated the assumptions of ANCOVA, we compared the output to robust linear models constructed using the lmRob function from the ‘robust’ package^63^. There were no differences between analyses performed with robust linear models and ANCOVAs.

Principle component analysis (PCA) was conducted using R base-package (R core team, 2014) and cluster analysis was conducted using the Mclust function from the “mclust” library^64^, which uses Baysian Information Criterion (BIC) scores from model-based inferences to calculate the optimum number of clusters. Data were normalised prior to PCA to ensure equal variance amongst groups.

Custom contrast matrices were used to make post-hoc multiple pair-wise comparisons of ANCOVAs with the estimable function from the ‘gmodels’ package^65^. All statistics were calculated using R v.3.1.2.

## Additional Information

**Data availability:** All original data from this manuscript is deposited on Dryad, and can be found at doi: 10.5061/dryad.3bd65.

**How to cite this article**: Perl, C. D. and Niven, J. E. Colony-Level Differences in the Scaling Rules Governing Wood Ant Compound Eye Structure. *Sci. Rep.* **6**, 24204; doi: 10.1038/srep24204 (2016).

## Supplementary Material

Supplementary Information
