# Supplementary material for: Colony-Level Differences in the Scaling Rules Governing Wood Ant Compound Eye Structure
Source: Sci Rep. 2016 Apr 12;6:24204. doi: 10.1038/srep24204 (PMC4828647; doi:10.1038/srep24204)
Supplement: Supplementary Information [file srep24204-s1.doc]

**Colony-Level Differences in the Scaling Rules Governing Wood Ant Compound Eye Structure**

**Craig D. Perl and Jeremy E. Niven**

**^1^School of Life Sciences and Centre for Computational Neuroscience and Robotics, University of Sussex, Falmer, Brighton, BN1 9QG, UK**

**Author for correspondence:**

Jeremy E. Niven

e-mail: J.E.Niven@sussex.ac.uk

**Supplemental Materials**

**
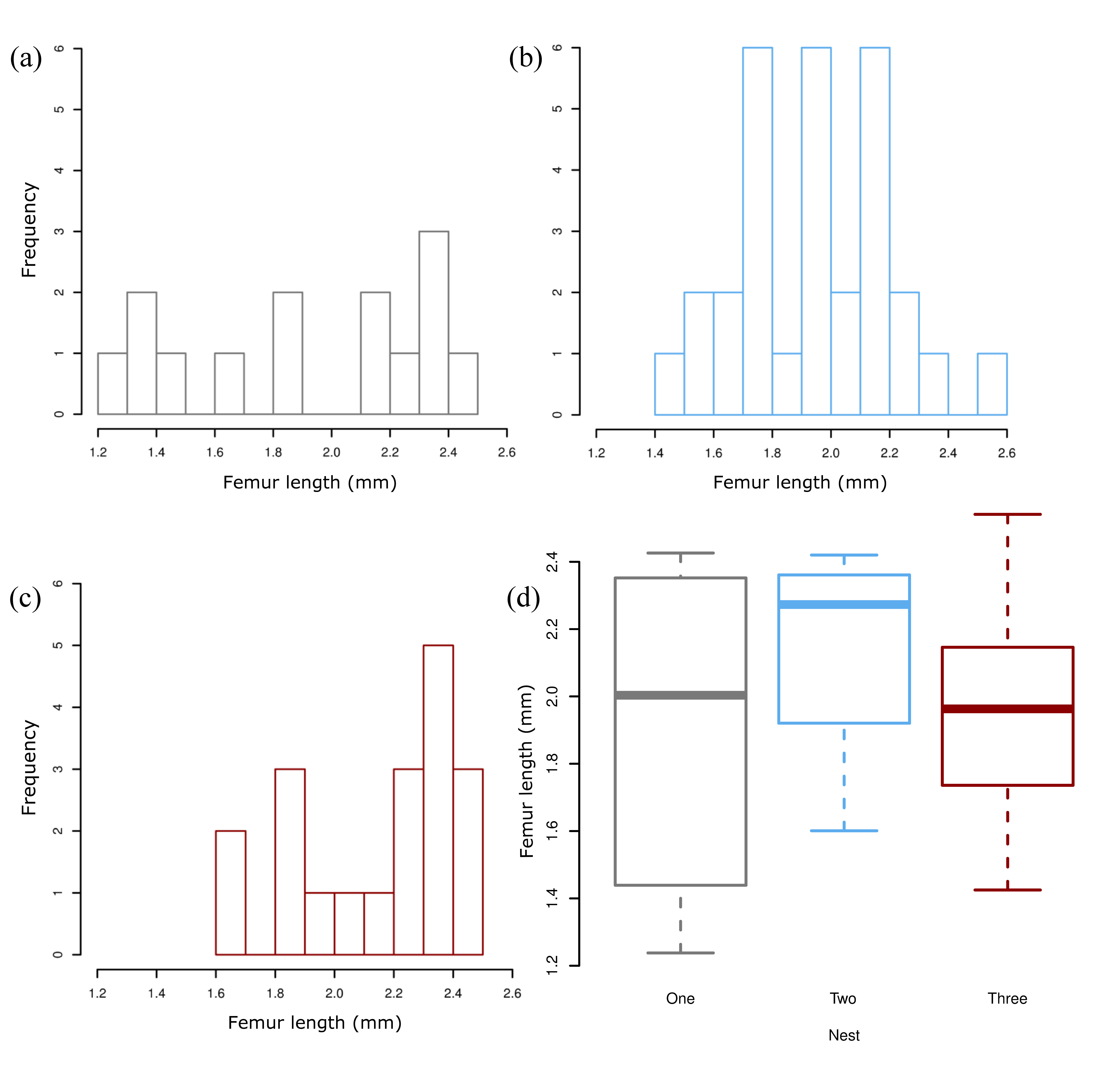
**

**Figure S1.** The size range and distribution of workers from three nests used in the investigation assessed by femur length. (a) Nest #1 (number of ants, n = 17), (b) Nest #2 (n = 29), (c) Nest #3 (n = 19), and (d) Range of femur length distributions from all three nests.


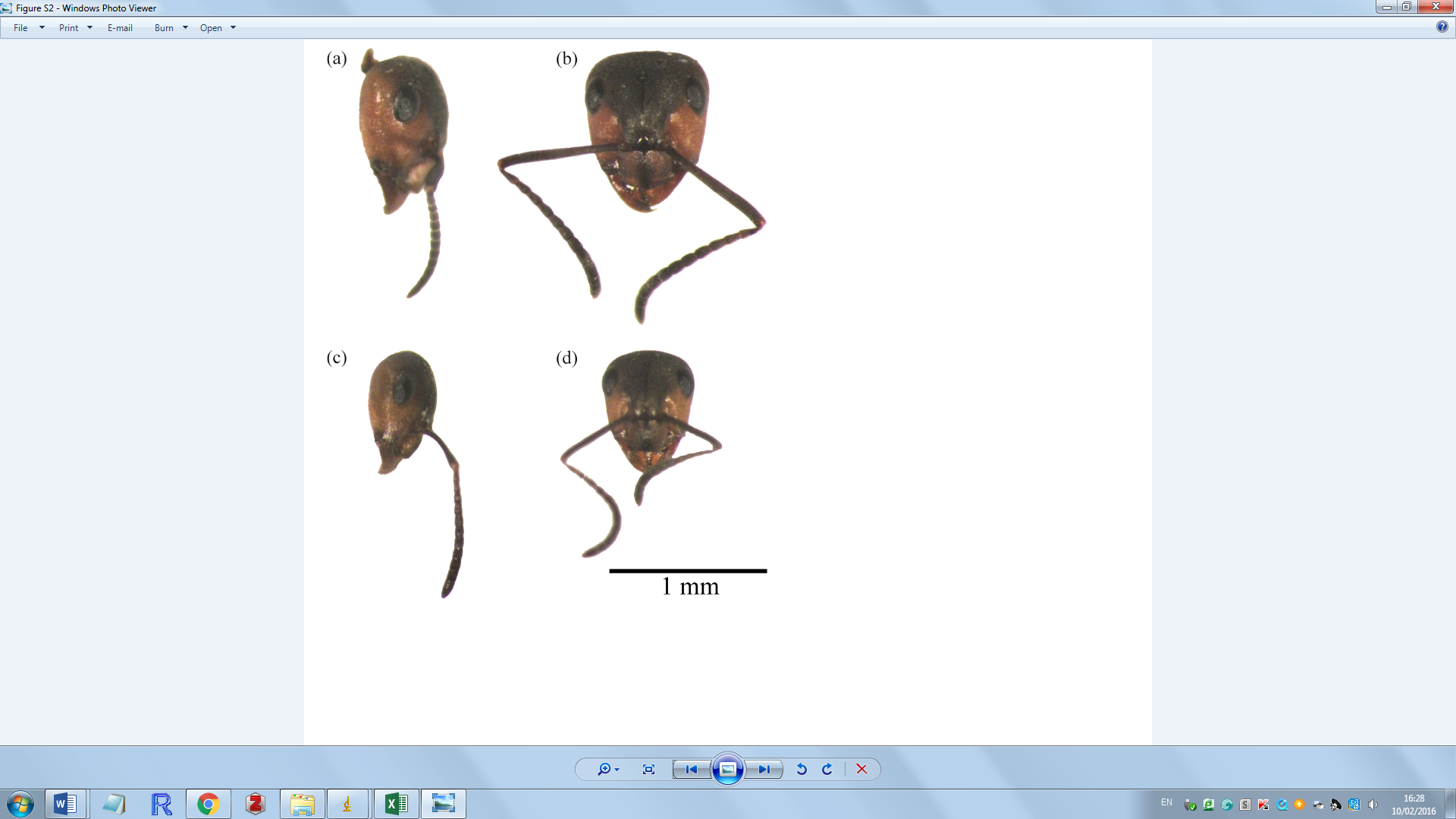


**Figure S2.** Differences in the head size of large and small workers. Lateral (*a*) and frontal (*b*) view of large ant worker head. (*c, d*) As in (*a*) and (*b*) but for the head of a smaller worker.

**
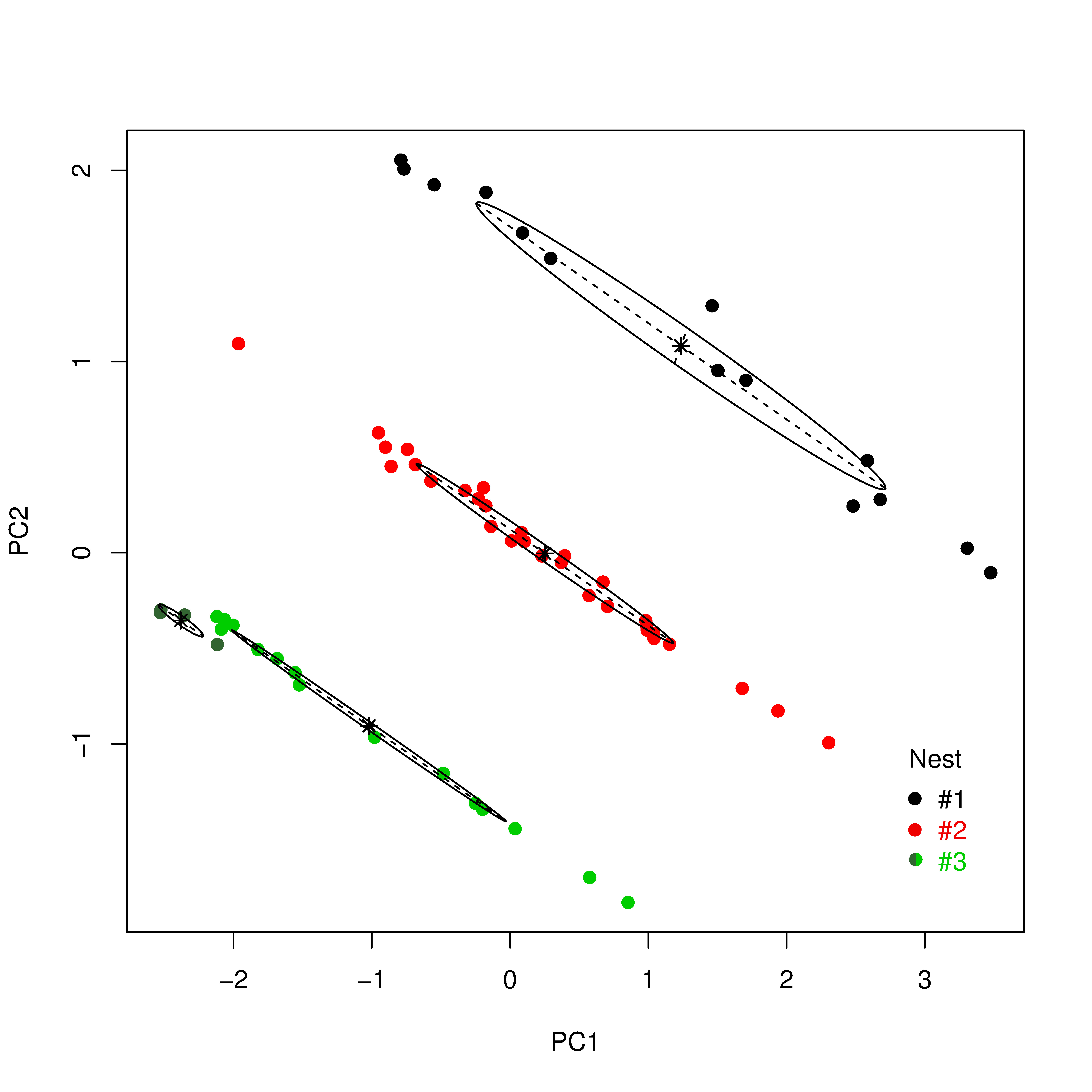
**

**Figure S3.** Cluster analysis of femur length, eye area and nest after dimension reduction using Principle Component Analysis (PCA).


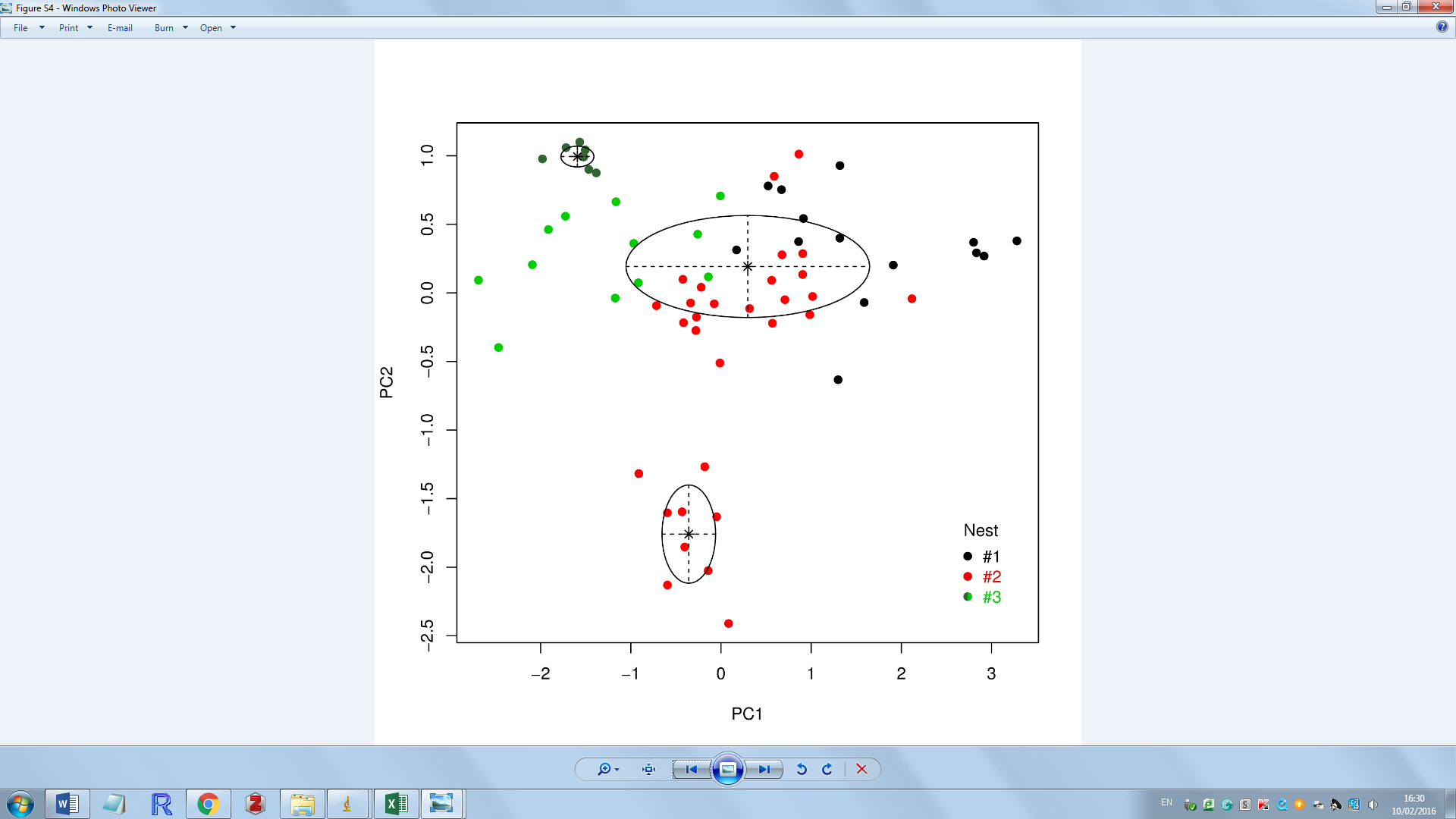


**Figure S4.** Cluster analysis of facet count, mean facet diameter and nest after dimension reduction using PCA.

**
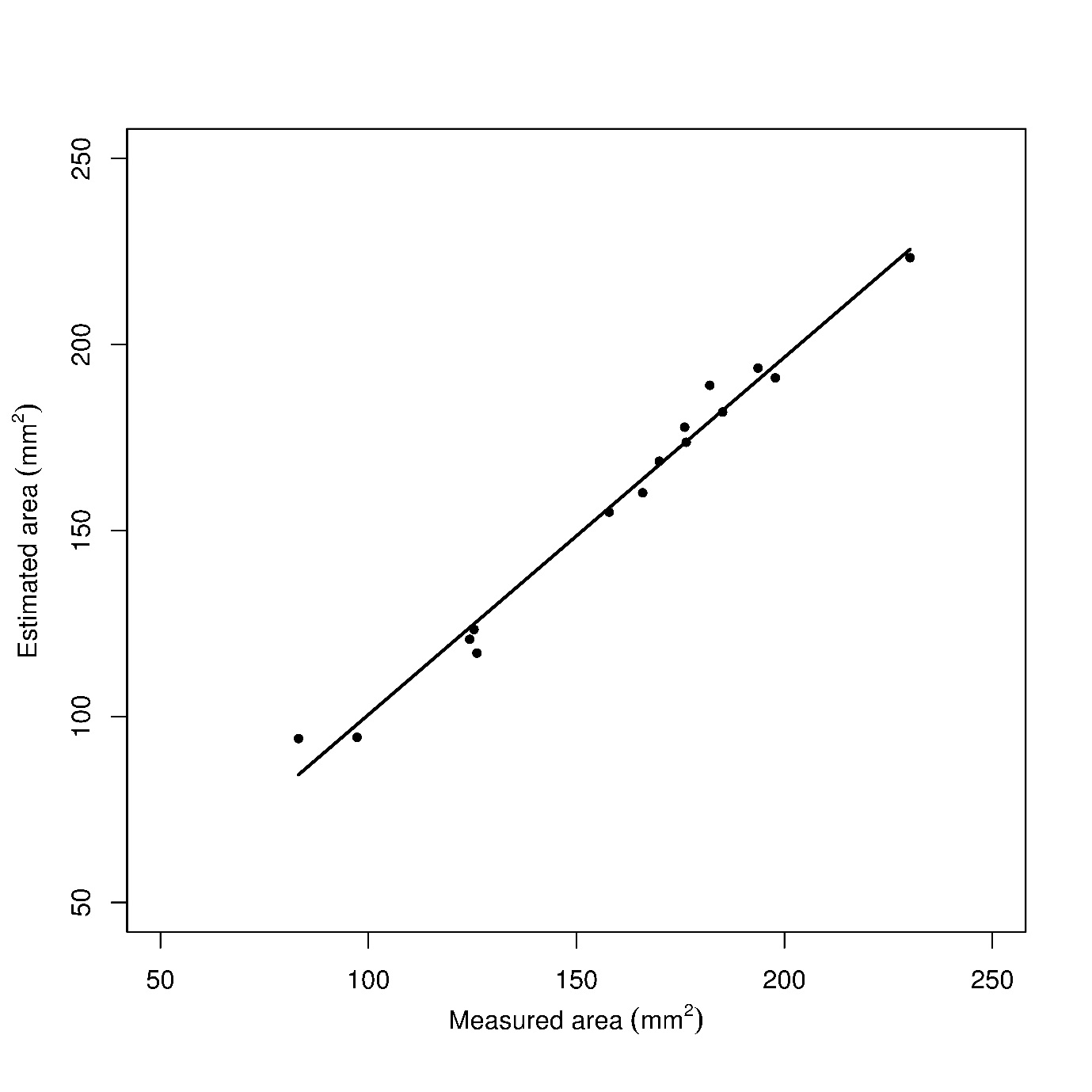
Figure S5.** Eye area is well approximated by an oval. The area of 15 ant eyes (5 from each nest) estimated by approximating the eye as an oval as a function of area of the same eyes measured with ImageJ. (Intercept = 4.39 ± 5.62, p = 0.448; slope = 0.96 ± 0.03, p < 0.0001; r^2^ = 0.98).

**Table S1.** Scaling co-efficients from gross morphological eye measurements. The co-efficients were calculated by fitting a linear model to log transformed data.

| **Nest** | **N** |  | | | **Parameter** | | **Slope (α)** | **±standard error** | | **Intercept log(b)** | | **±standard error** | |  |
| --- | --- | --- | --- | --- | --- | --- | --- | --- | --- | --- | --- | --- | --- | --- |
| #1 | 14 | |  | Eye area | | 0.56 | | | 0.12 | | 2.01 | | 0.08 | |
| #2 | 30 | |  | Eye area | | 0.68 | | | 0.05 | | 2.03 | | 0.03 | |
| #3 | 19 | |  | Eye area | | 0.72 | | | 0.06 | | 2.04 | | 0.06 | |
| #1 | 14 | |  | Facet diameter | | 0.19 | | | 0.03 | | 2.66 | | 0.05 | |
| #2 | 30 | |  | Facet diameter | | 0.36 | | | 0.07 | | 2.63 | | 0.05 | |
| #3 | 19 | |  | Facet diameter | | 0.19 | | | 0.06 | | 2.72 | | 0.05 | |
| #1 | 14 | |  | Facet number | | 0.91 | | | 0.04 | | 5.80 | | 0.03 | |
| #2 | 30 | |  | Facet number | | 0.52 | | | 0.17 | | 6.02 | | 0.11 | |
| #3 | 19 | |  | Facet number | | 0.96 | | | 0.06 | | 5.86 | | 0.05 | |

| **Variable** | **Component 1** | **Component 2** | **Component 3** |
| --- | --- | --- | --- |
| Femur length | -0.611 | -0.461 | 0.644 |
| Eye area | -0.660 | -0.152 | -0.736 |
| Nest | -0.437 | 0.874 | 0.211 |
| Facet count | -0.579 | 0.568 | 0.585 |
| Mean facet diameter | -0.534 | -0.806 | 0.254 |
| Nest | -0.616 | 0.165 | -0.770 |

**Table S2.** Loadings from principle component analysis used as a precursor to the cluster analysis. Loadings were used for examining the effects of nest, eye area and femur or the effects of nest, mean facet diameter and facet number.
